# Preparation of Glutathione-Responsive Paclitaxel Prodrug Based on Endogenous Molecule of L-Glutathione Oxidized for Cancer Therapy

**DOI:** 10.3390/pharmaceutics16091178

**Published:** 2024-09-06

**Authors:** Xiao Duan, Qiang Wang, Yue Wang, Xinping Liu, Manman Lu, Zhifang Li, Xuelian Jiang, Jingquan Ji

**Affiliations:** 1 Changzhi Key Laboratory of Drug Molecular Design and Innovative Pharmaceutics, Shanxi Provincial Department-Municipal Key Laboratory Cultivation Base for Quality Enhancement and Utilization of Shangdang Chinese Medicinal Materials, School of Pharmacy, Changzhi Medical College, Changzhi 046000, China; wangqiang990326@163.com (Q.W.); xp99714@163.com (X.L.); 19834819324@163.com (Z.L.); 17860396873@163.com (X.J.); 2 The Stem Cell and Tissue Engineering Research Center, Changzhi Medical College, Changzhi 046000, China; 3 Central Lab Changzhi Medical College, Changzhi Medical College, Changzhi 046000, China; 17696157396@163.com (Y.W.); 13383453636@163.com (M.L.)

**Keywords:** glutathione responsiveness, paclitaxel prodrug, micelles, antitumor

## Abstract

Using an endogenous carrier is the best method to address the biocompatibility of carriers in the drug delivery field. Herein, we prepared a glutathione-responsive paclitaxel prodrug micelle based on an endogenous molecule of L-glutathione oxidized (GSSG) for cancer therapy using one-pot synthesis. The carboxyl groups in L-glutathione oxidized were reacted with the hydroxyl group in paclitaxel (PTX) using the catalysts dicyclohexylcarbodiimide (DCC) and 4-dimethylaminopyridine (DMAP). Then, the amino-polyethylene glycol monomethyl ether (mPEG-NH_2_) was conjugated with GSSG to prepare PTX-GSSG-PEG. The structure of PTX-GSSG-PEG was characterized using infrared spectroscopy (FT-IR), nuclear magnetic resonance spectroscopy (NMR), and mass spectrometry (MS). The drug release kinetics of PTX within PTX-GSSG-PEG were quantified using ultraviolet spectroscopy (UV-Vis). The size of the PTX-GSSG-PEG micelles was 83 nm, as evaluated using dynamic light scattering (DLS), and their particle size remained stable in a pH 7.4 PBS for 7 days. Moreover, the micelles could responsively degrade and release PTX in a reduced glutathione environment. The drug loading of PTX in PTX-GSSG-PEG was 13%, as determined using NMR. Furthermore, the cumulative drug release rate of PTX from the micelles reached 72.1% in a reduced glutathione environment of 5 mg/mL at 120 h. Cell viability experiments demonstrated that the PTX-GSSG-PEG micelles could induce the apoptosis of MCF-7 cells. Additionally, cell uptake showed that the micelles could distribute to the cell nuclei within 7 h. To sum up, with this glutathione-responsive paclitaxel prodrug micelle based on the endogenous molecule GSSG, it may be possible to develop novel nanomedicines in the future.

## 1. Introduction

Paclitaxel (PTX) is a diterpenoid compound extracted from the bark of the Taxus chinensis tree [1,2]. It acts on microtubule proteins via non-covalent interactions to inhibit tumor growth [2]. This potent antitumor drug is mainly used against breast cancer, ovarian cancer, and lung cancer in hospitals. Unfortunately, the poor water solubility, low bioavailability, and severe side effects of PTX derivatives on normal tissues limit its clinical use [3,4]. Although the PTX-related pharmaceuticals of the PTX–Albumin nanoparticles (Abraxane) and liposomal PTX on the market could increase its water solubility and bioavailability to some degree, the severe side effects remain unresolved [5].

Addressing the severe side effects and water solubility of PTX derivatives is a major aim in the development of better PTX-related pharmaceuticals. PTX-based prodrugs have been prepared to address the above issues [6,7,8]. In order to speed up the clinical application of PTX derivative-based prodrugs, researchers usually select the Food and Drug Administration (FDA)-approved excipient of polyethylene glycol (PEG) to prepare prodrugs [9,10,11,12,13,14], such as PEGylated lithocholic acid (LCA)-docetaxel (DTX) conjugate [9] and hypoxia-responsive [10] and oxidation-responsive PEGylated PTX prodrugs [14]. These PEGylated PTX prodrugs usually need some linkers to conjugate with PTX. Unfortunately, the linkers have not been approved by the FDA because their biocompatibility and biodegradability have not been fully evaluated in vivo. For example, Tian et al. used 4,4’-Dithiodibutyric acid [15] and Ma et al. used 3,3′-dithiodipropionic acid [16] as linkers to synthesize PTX prodrugs. Meanwhile, some non-responsive endogenous linkers of docosahexaenoic acid [17] and peptides [18] have been used to prepare PTX-based prodrugs.

Based on the above summary of linkers, we selected a better linker of L-glutathione oxidized (GSSG) to prepare a PTX-based prodrug. GSSG is an endogenous molecule in human beings [19,20]. The disulfide bond in its structure could be controllably broken in tumor tissue with high-concentration reduced glutathione (GSH) [21,22,23]. The GSH concentrations in different tumor cells, including pancreatic cancer [24], human lung adenocarcinoma (A549) [25], liver cancer (HepG2) [26], cervical cancer (Hela) [26], and breast cancer (MCF-7) [26] cells, are higher than in normal cells. Therefore, the linker of GSSG with GSH responsiveness and biocompatibility is a better choice for preparing PTX-based prodrugs than the abovementioned linkers in the drug delivery field for cancer therapy.

Herein, a GSH-responsive PTX prodrug micelle was prepared with the endogenous molecule GSSG as the linker for breast cancer therapy. Highly water-soluble GSSG could significantly increase the water solubility and decrease the side effects of PTX. Meanwhile, the disulfide bond in GSSG could be broken under a high concentration of GSH in tumor tissues, releasing PTX for cancer therapy. Together, the results demonstrated that this GSH-responsive prodrug micelle based on the endogenous molecule GSSG will significantly improve water solubility and provide a new linker for developing PTX-related pharmaceuticals in the future.

## 2. Materials and Methods

### 2.1. Materials

Paclitaxel (PTX, purity: 99%), 4-dimethylaminopyridine (DMAP, purity: 99%), and dialysis bags (MWCO = 8000 Da and MWCO = 3500 Da) were supplied by the Aladdin company. 1-hydroxybenzotriazole (HOBt, purity: 99%) was provided by bidepharm. N,N-dicyclohexylcarbodiimide (DCC, purity: 99%) was purchased from Shanghai Haohong Biochemical Technology Co., Ltd. (Shanghai, China). L-glutathione oxidized (GSSG, purity: 98%) was supplied by Damas-Beta. mPEG-NH_2_ (MW = 5000) was purchased from the Yarebio company (Shanghai, China). Reduced glutathione (GSH) was supplied by Shanghai Dingxian Biochemical Technology Co., Ltd. (Shanghai, China). Dulbecco’s Modified Eagle Medium (DMEM) was supplied by Beijing Solarbio Science. Fetal bovine serum was purchased from Zhejiang Tianhang Biotechnology Co., Ltd (Zhejiang, China). 1,1′-dioctadecyl-3,3,3′,3′-tetramethylindodicarbocyanine,4-chlorobenzenesulfonate salt (DID), DID-cell membrane far-infrared fluorescent probes, Hoechst staining kits, penicillin-streptomycin solution (100×), trypsin (0.25%), phosphate-buffered saline (PBS), Hoechst 33258, antifade mounting medium, Annexin V-FITC cell apoptosis detection kits and cell counting kit-8 (CCK-8), and confocal culture dishes (BeyoGold™ 35 mm) were purchased from Beyotime Biotechnology Company. MCF-7 cells were purchased from Wuhan Shangen Biotechnology Co., Ltd (Wuhan, China). An ultra-centrifugal filter was purchased from Shanghai Wishes Biotechnology (Millipore, MWCO = 10 KD, Shanghai, China). All biological reagents or kits were used according to the protocols of Beyotime Biotechnology Company (Shanghai, China). All other chemical and organic solvents were directly used without further purification.

### 2.2. Methods

The structure of PTX-GSSG-PEG was confirmed using FT-IR (Bruker, Berlin, Germany), NMR (400 MHz, Bruker AVANCE NEO, Germany), and MS (Bruker microflex LT/LRF, Germany). The elute time was measured using gel permeation chromatography with a 1 mL/min flow rate at 35 °C in THF (Agilent 1260, Refractive Index, polystyrene standard). The particle sizes of PTX-GSSG-PEG (1 mg/mL) were measured using TEM (FEI Tecnai F20, voltage: 200 kv; Oregon, USA) and DLS (Nano ZSE, Malvern, UK; Laser: 50 mM, 532 nm) in PBS. The micelles (50 μL) were dripped onto copper mesh without any staining agent. The drug release rate was determined using UV-Vis (Shimadzu, Kyoto, Japan). Cell viability and hemolysis were measured using a microplate reader at 450 nm and 540 nm, respectively. The distribution of micelles in cells was evaluated using confocal laser scanning microscopy (CLSM, LEICA/SP8, Wetzlar, Germany). Cell viabilities were estimated using a CCK-8 assay.

### 2.3. Synthesis and Characterization of PTX-GSSG-PEG

Paclitaxel (PTX, 0.1 mmol, 85 mg), L-glutathione oxidized (GSSG, 0.1 mmol, 61 mg), dicyclohexylcarbodiimide (DCC, 0.1 mmol, 21 mg), and 4-dimethylaminopyridine (DMAP, 0.025 mmol, 3 mg) were placed in a round-bottom flask with 10 mL of N, N-dimethylformamide (DMF) to react for 24 h in a 40 °C water bath. Then, mPEG-NH_2_ (0.1 mmol, 500 mg), dicyclohexylcarbodiimide (DCC, 0.1 mmol, 21 mg), and 1-hydroxybenzotriazole (HOBt, 0.1 mmol, 13 mg) were added to the round-bottom flask to react for another 24 h. Afterward, the reaction solution was placed in a dialysis bag (MWCO = 8000) and immersed in a beaker with fresh DMF for 1 day. The DMF was replaced with fresh DMF thrice in 1 day. After that, the DMF in the beaker was replaced with fresh water thrice. Finally, the micelle solution in the dialysis bag was taken out and freeze-dried in a lyophilizer to obtain PTX-GSSG-PEG powder.

PTX, GSSG, and PTX-GSSG-PEG were dissolved in an NMR tube with DMSO-d_6_, and the NMR spectra were measured using a Bruker AVANCE NEO. The powders of PTX, GSSG, mPEG-NH_2_, and PTX-GSSG-PEG were mixed with KBr to prepare tablets for measuring the FT-IR spectra. The UV-Vis spectra of PTX and PTX-GSSG-PEG were measured in methanol. The MS spectra were measured by SCI-GO company (Beijing City) in China.

### 2.4. Preparation of PTX-GSSG-PEG Micelles

PTX-GSSG-PEG (10 mg) was dissolved in DMSO (200 μL); then, the DMSO solution was dripped into water (10 mL) in a vial over 30 min with magnetic stirring (1000 rpm) to prepare the micelles. To remove the DMSO (200 μL), the micelle solution was filtered by using an ultra-centrifugal filter at 3500 rpm by adding PBS 8 times (5 mL/per time). The final micelles were prepared to measure the particle size using DLS and TEM. In addition, the DID solution (20 μL, 5 mg/mL in DMSO) was added to DMSO (200 μL). Then, DID@PTX-GSSG-PEG was prepared using the same process.

### 2.5. Drug Loading and Release In Vitro

The drug loading was determined by using the NMR and MS spectra of PTX-GSSG-PEG according to the integrals of characteristic peaks and molecular weight.

PTX-GSSG-PEG (10 mg) was dissolved in 10 mL of pH 7.4 PBS to directly form micelles and was equally divided into two dialysis bags (MWCO = 3500). Then, one dialysis bag was placed in a flask including 45 mL of pH 7.4 PBS with 5 mg·mL^−1^ of GSH, and the other was placed in a flask with 45 mL of pH 7.4 PBS. Afterward, the two flasks were placed in a constant-temperature (37 °C) water bath shaker (80 rpm/min). During the drug release process, the solution (200 μL) outside the dialysis bag in the flask was taken out to measure the drug accumulative rate at a fixed time point. The time points were set to 0.5 h, 1 h, 2 h, 24 h, 72 h, 120 h, and 168 h. After the final time point, the solution in the dialysis bag was mixed with the solution outside the dialysis bag; then, the total drug in the mixed solution was measured by UV-Vis at 230 nm according to the standard curve of PTX. The experiment was conducted twice.

### 2.6. Cell Viability and Flow Cytometry Experiments

The MCF-7 cell suspension was seeded in a 96-well plate (8000 cells/well) for 12 h. Then, the supernatant was discarded and the culture media with PTX–Albumin and PTX-GSSG-PEG micelles were added to the wells (200 μL/well). The concentrations of PTX–Albumin and PTX-GSSG-PEG (PTX concentration) were set to 0 μg·mL^−1^, 5 μg·mL^−1^, 10 μg·mL^−1^, 20 μg·mL^−1^, 40 μg·mL^−1^, and 60 μg·mL^−1^. After 24 h and 48 h, the old culture media in the 96-well plate were replaced with fresh PBS; then, the CCK-8 staining solution (20 μL) was added to each well. One hour later, the 96-well plate was measured under a microplate reader at 450 nm. Samples were performed in duplicate (n = 6).

The MCF-7 cell suspension was seeded in a 6-well plate for 12 h. Then, the PTX–Albumin and PTX-GSSG-PEG micelles (PTX concentration: 2.5 μg·mL^−1^, 5 μg·mL^−1^, and 20 μg·mL^−1^) with fresh media were added to wells for 24 h. The PI and Annexin V-FITC apoptosis kits were used to measure the cell apoptosis rate. The protocol of the apoptosis kits was obtained from Beyotime Biotechnology Company.

### 2.7. Cellular Uptake Experiment

MCF-7 cells were seeded in confocal culture dishes (5 × 10^4^ cells/well) and incubated in a CO_2_ incubator at 37 °C for 12 h. Afterward, the culture media were removed and fresh culture media with DID (1 μg·mL^−1^) or DID@PTX-GSSG-PEG (100 μg·mL^−1^) micelles were added to the dishes. Then, the cells in the dishes were observed under CLSM at 2 h, 4 h, and 7 h. The specific process was as follows: The old media were discarded, and the cells were rinsed with PBS and fixed using Carnoy’s fix solution for 10 min at room temperature. Subsequently, the cell nuclei were stained with Hoechst 33258 for 5 min, then, the cells were rinsed with sterile PBS thrice. Finally, the antifade mounting medium was added to the confocal dishes to obtain the final samples for monitoring the distribution of micelles in the MCF-7 cells at 2 h, 4 h, and 7 h.

### 2.8. Hemolysis Test

Whole blood (2.0 mL) was collected from rats and prepared for a mixed-cell suspension with erythrocytes. The mixed-cell suspension in PBS (8 mL) was equally divided into 8 tubes. Then, the tubes were centrifuged to obtain the mixed cells. Subsequently, (1) pure water, (2) a 0.9% NaCl solution, (3) PTX–Albumin (including 20 μg·mL^−1^ of PTX), and (4) PTX-GSSG-PEG micelles (including 20 μg·mL^−1^ of PTX) were added to the tubes with the mixed cells and placed in an incubator with 5% CO_2_ at 37 °C for 30 min. Finally, these tubes were centrifuged at 1000 rpm for 3 min to observe the hemolysis phenomenon. Moreover, the supernatants (100 μL) were collected and added to a 96-well plate in order to measure the absorbance under a microplate reader at 540 nm.

## 3. Results and Discussion

### 3.1. Characterization of PTX-GSSG-PEG

**Figure 1:**
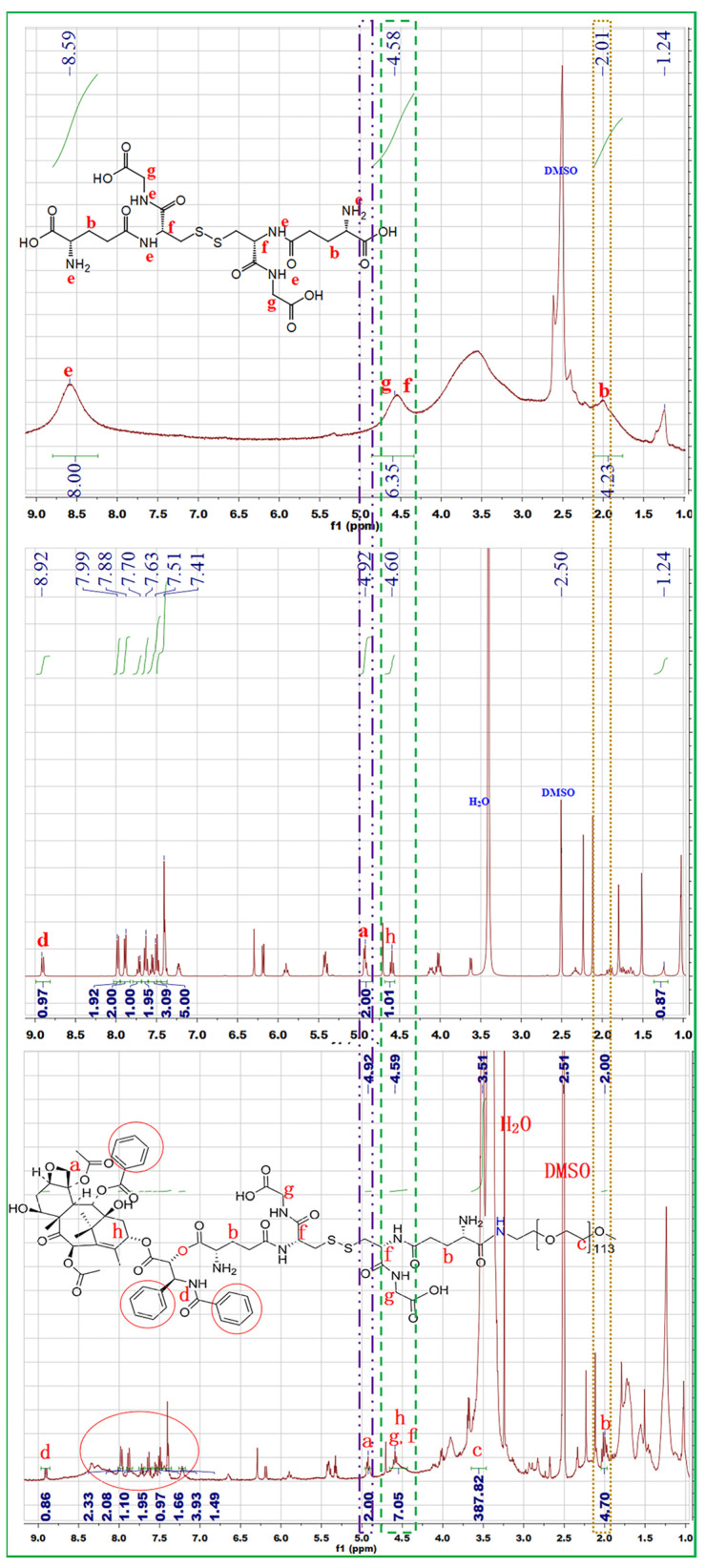
The NMR spectra of GSSG (**top**), PTX (**middle**), and PTX-GSSG-PEG (**bottom**) in DMSO-d_6_.

**Scheme 1:**
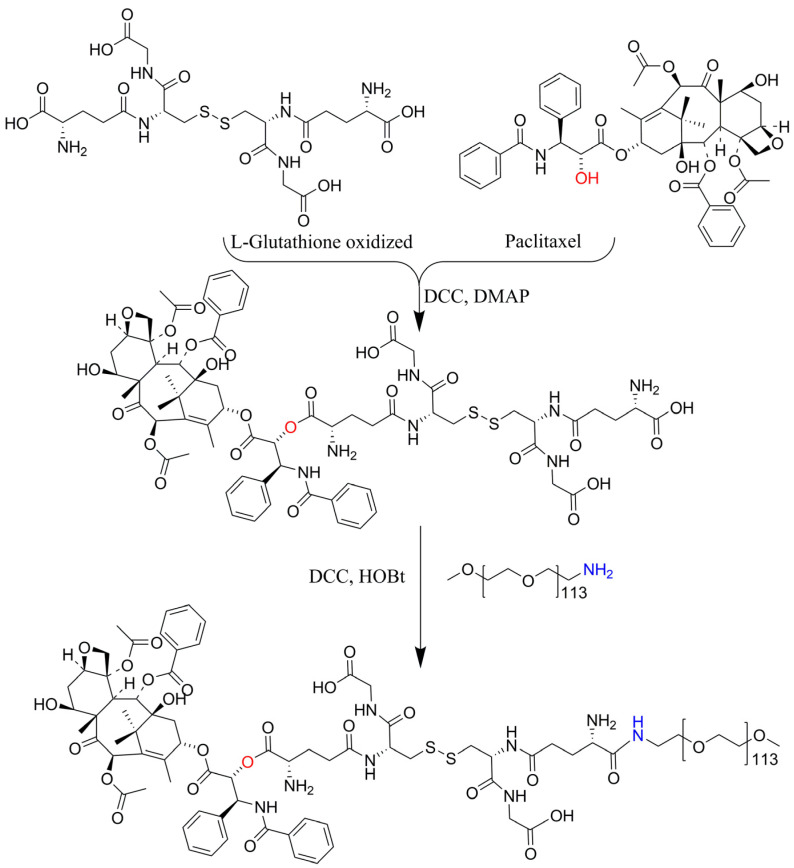
The synthesis route of PTX-GSSG-PEG.

**Figure 2:**
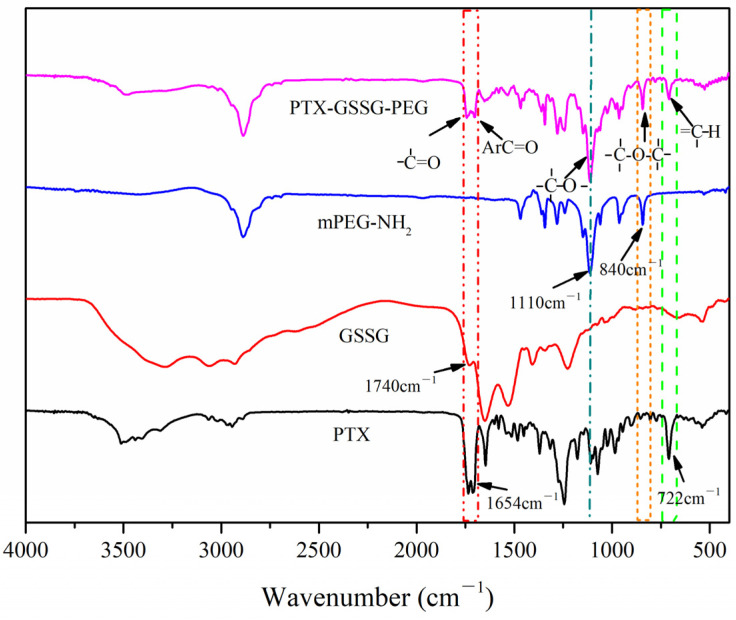
The FT-IR spectra of PTX, GSSG, mPEG-NH_2_, and PTX-GSSG-PEG.

The synthesis route of PTX-GSSG-PEG is shown in Scheme 1. The structure of PTX contains hydroxyls. The COOH group in GSSG was reacted with OH in PTX with the catalysts DCC and DMAP. Afterward, mPEG-NH_2_ was reacted with COOH in GSSG. During the reaction, PTX was reacted with GSSH first in case the yield of PTX-GSSG-PEG decreased. The optimal mass ratio of PTX, GSSG, and PEG_5000_ was 1:1:1. Based on the mass ratio, we calculated the theoretical drug loading rate to be 13%. To confirm the structure of PTX-GSSG-PEG, we compared the NMR spectra of PTX, GSSG, and PTX-GSSG-PEG (Figure 1). The characteristic peaks of PTX (a position, 4.92 ppm), GSSG (b position, 2.00 ppm), and PEG (c position) were simultaneously observed in the spectrum of PTX-GSSG-PEG. Meanwhile, the ratio of PTX, GSSG, and PEG could be obtained according to the characteristic integrals in the PTX-GSSG-PEG spectrum. The integral numbers of positions a (PTX), b (GSSG), and c (PEG) were 2.00, 4.70, and 387.82 in the PTX-GSSG-PEG spectrum, respectively. Based on the integrals of the characteristic peaks in the PTX-GSSG-PEG spectrum, we calculated the ratio of PTX, GSSG, and PEG to be approximately 1:1:1. Moreover, the MS spectrum (Figure S1) showed that molecular weight of PTX-GSSG-PEG was between 4700 and 6600, thus confirming that PTX-GSSG-PEG included one molecule of PTX, GSSG, and PEG_5000_. Furthermore, according to the elute times of PEG (16.330 min) and PTX-GSSG-PEG (16.305 min) measured using GPC (Figure S2), we speculated that PTX or GSSG conjugated with PEG. To confirm the exact reactive sites of the carboxyl groups in GSSG, we analyzed the g position in GSSG. We found that the integrals of the g (including the f position) and h positions were 6 and 1, respectively. In the PTX-GSSG-PEG NMR spectrum, we calculated the total integrals of g, h, and f to be 7.05 based on the integral of the a position with two protons (two protons in the a position indicate one PTX molecule). These integral numbers of the g (four protons), f (two protons), and h (one proton) positions confirmed that the four protons in the g position in GSSG did not shift to any other position. This means that the carboxyl groups adjacent to the g position in PTX-GSSG-PEG did not react with PTX or PEG (Figure 1). Moreover, it has been demonstrated that, regarding the reactive activity of α-COOH and β-COOH, only α-COOH could be activated by N-phosphorylation under mild conditions [24]. The reactive activity of the carboxyl groups in GSSG in this study could perhaps be explained by peptide synthesis (α-COOH prefers to react with another amino acid) in vivo according to the above reference. Additionally, the characteristic peaks in the PTX (722 cm^−1^) and PEG (840 cm^−1^ and 1110 cm^−1^) FT-IR spectra were also observed in the PTX-GSSG-PEG spectra (Figure 2). These results demonstrated that PTX-GSSG-PEG was successfully synthesized.

### 3.2. Drug Loading and Release

The drug loading of PTX was determined via the NMR and MS spectra of PTX-GSSG-PEG. According to the integrals of the characteristic peaks and the molecular weight of PTX-GSSG-PEG (Figure 1 and Figure S1), there was one molecule of PTX, GSSG, and mPEG-NH_2_ in PTX-GSSG-PEG. Hence, the drug loading rate of PTX in PTX-GSSG-PEG was 13% according to the molecular weight of PTX, GSSG, and PEG.

**Figure 3:**
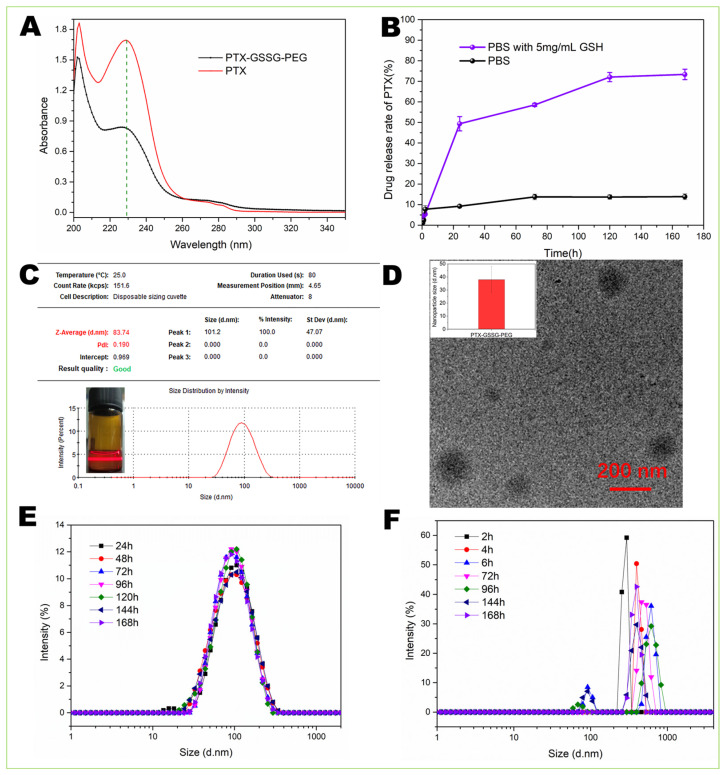
(**A**) Spectra of PTX and PTX-GSSG-PEG measured using UV-Vis. (**B**) Drug release rate of PTX from PTX-GSSG-PEG during 168 h. Particle size of PTX-GSSG-PEG micelles measured using (**C**) DLS and (**D**) TEM. (**E**) Stability without GSH and (**F**) GSH responsiveness in 5 mg/mL of GSH pH 7.4 PBS measured using DLS.

The drug release rate of PTX was measured using UV-Vis (Figure 3A). Because the UV curves of PTX and PTX-GSSG-PEG in methanol were similar to the UV curve of PTX-GSSG-PEG in PBS, we used the standard equation of PTX in methanol to calculate the drug release rate. The PTX release rate from the micelles in 5 mg/mL of GSH was 72.1% and 75% at 120 h and 168 h, respectively (Figure 3B). The slight difference between the rates at 120 h and 168 h could be attributed to the low diffusion rate after 120 h with a concentration gradient-driven drug release mode. At the same time, PTX could not be released from PTX-GSSG-PEG in PBS without GSH, confirming that GSH-responsive PTX-GSSG-PEG could controllably release PTX in a high concentration of GSH in tumor cells [25,26].

### 3.3. Particle Size and Stability of Micelles

PTX-GSSG-PEG was assembled into micelles in PBS. The particle size of the PTX-GSSG-PEG micelles measured using DLS was around 83 nm, with a narrow PDI (0.19) (Figure 3C). The TEM result showed that the assembled micelles were spherical and that their average size was 37 nm (Figure 3D). To evaluate the stability of the PTX-GSSG-PEG micelles in pH 7.4 PBS, we measured the particle changes in the micelles during 7 days at room temperature without any inertia atmosphere. The results demonstrated that the PTX-GSSG-PEG micelles were stable in PBS without GSH (Figure 3E). As a comparation group, we measured the particle changes in PTX-GSSG-PEG micelles in PBS with 5 mg/mL of GSH. The PTX-GSSG-PEG micelles were disrupted after 2 h, with a higher polydisperse index (1.00) (Figure 3F). This GSH-responsive disruption of micelles demonstrates that the PTX release rate is reliable, as shown in Figure 3B.

### 3.4. Cell Viability and Flow Cytometry Experiments

**Figure 4:**
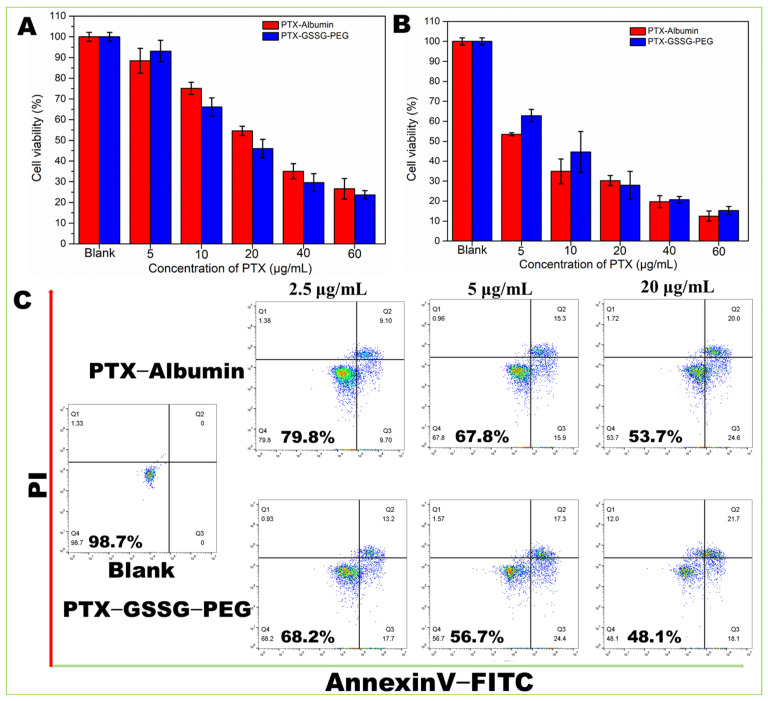
The MCF-7 cell viabilities of PTX–Albumin and PTX-GSSG-PEG with different concentrations of PTX at (**A**) 24 h and (**B**) 48 h evaluated using a CCK-8 assay. (**C**) MCF-7 cell apoptosis triggered by PTX–Albumin and PTX-GSSG-PEG with different concentrations of PTX at 24 h.

Because of the low water solubility of free PTX, we could not dissolve it in culture media to evaluate cell viability. To evaluate the drug efficacy of PTX-GSSG-PEG, we chose a PTX pharmaceutical (PTX–Albumin) as the control group. In the CCK-8 assays, we found that the PTX-GSSG-PEG micelles had a similar drug efficacy to PTX–Albumin at 24 h (Figure 4A) and 48 h (Figure 4B). The IC_50_ values of PTX–Albumin and the PTX-GSSG-PEG micelles were 27 μg/mL and 18 μg/mL at 24 h, respectively. Meanwhile, we evaluated the apoptosis of MCF-7 cells using flow cytometry at 24 h. The results showed that the cell viabilities of the PTX-GSSG-PEG micelle group and the PTX–Albumin group (including 20 μg/mL PTX) were 48.1% and 53.7% at 24 h (Figure 4C), respectively. In addition, the early and late apoptosis rates were 24.6% and 20.0%, respectively, in the PTX–Albumin group with 20 μg/mL of PTX. In the PTX-GSSG-PEG micelle group, the early and late apoptosis rates were 18.1% and 21.7%, respectively. According to the results of the CCK-8 assays and cytometry experiments, this PTX-GSSG-PEG micelle had a similar drug efficacy to PTX–Albumin.

### 3.5. Cellular Uptake

**Figure 5:**
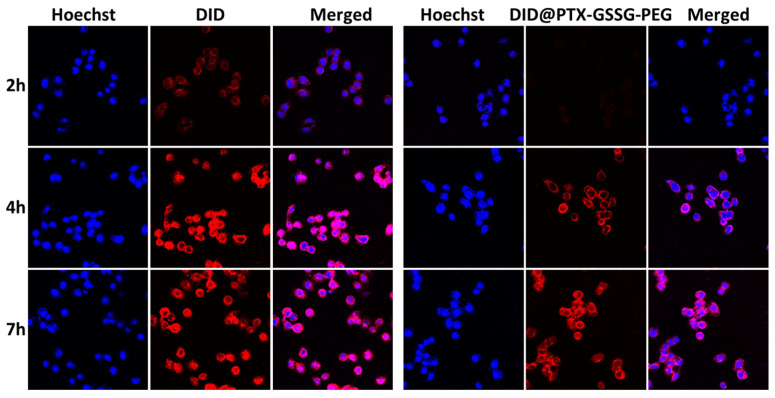
The distribution of DID and DID@PTX-GSSG-PEG at 2 h, 4 h, and 7 h measured using CLSM (scale: 40×; blue represents cell nuclei, and red represents DID).

To monitor the distribution of PTX-GSSG-PEG in tumor cells, we prepared DID@PTX-GSSG-PEG micelles and obtained a fluorescence image under the CLSM. In order to distinguish the free DID from DID@PTX-GSSG-PEG, we selected free DID as the control group. Figure 5 shows that the free DID (in red) overlapped the cell nuclei (in blue) at 4 h, while DID@PTX-GSSG-PEG did not distribute into the cell nuclei at 4 h. The blue color was surrounded by the red color in the DID@PTX-GSSG-PEG group at 4 h. The difference between the distribution behaviors of the free DID and DID@PTX-GSSG-PEG could be explained by the different uptake mechanisms. Small molecule drugs usually penetrate the cell membrane via free diffusion, while nanoparticles can be swallowed by cells via clathrin-coated pit-mediated endocytosis (CME) [27,28]. These results also demonstrate that PTX-GSSG-PEG could change the distribution of free DID when DID is encapsulated in micelles. Given that free DID indicates free PTX, the distribution differences between free DID (PTX) and DID@PTX-GSSG-PEG at 4 h confirmed that the PTX-GSSG-PEG nanomedicine based on PTX and GSSG could affect the distribution of free PTX in tumor cells. Although DID@PTX-GSSG-PEG distributed into the cell nuclei slower than free DID at 4 h, differences in the distribution between DID and DID@PTX-GSSG-PEG could not be distinguished at 7 h, confirming that PTX-GSSG-PEG is able to distribute into cell nuclei after 7 h.

### 3.6. Hemolysis Test

**Figure 6:**
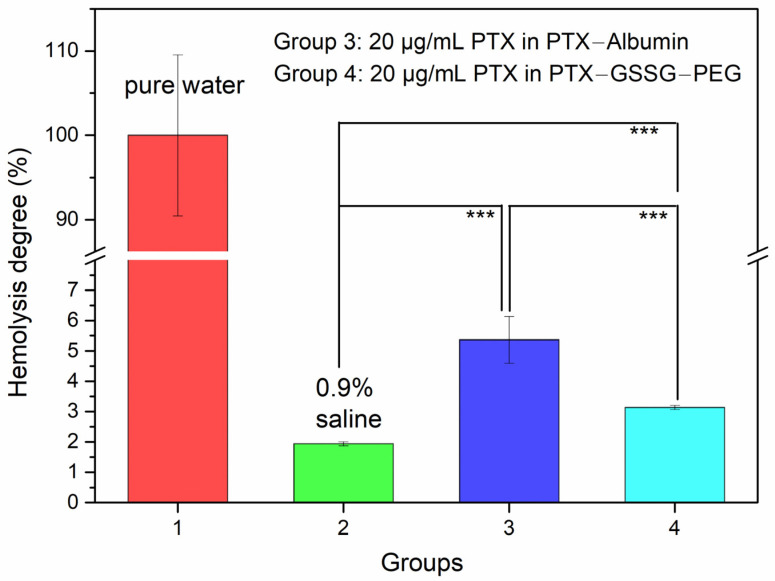
The hemolysis phenomena of pure water, 0.9% of saline, PTX–Albumin, and PTX-GSSG-PEG measured using a microplate reader at 540 nm in an incubator after 30 min (*** *p* < 0.001).

The hemolysis test is a common method used to evaluate the biocompatibility of biomaterials. Herein, we prepared a PTX–Albumin and PTX-GSSG-PEG solution to evaluate possible side effects on normal cells. Figure 6 shows that the hemolysis degree of PTX–Albumin (including 20 μg/mL of PTX; hemolysis degree: 5.4%) was significantly higher than that of the 0.9% saline group (hemolysis degree: 1.9%) and the PTX-GSSG-PEG (including 20 μg/mL of PTX; hemolysis degree: 3.1%) group. This significant difference between PTX–Albumin and the 0.9% saline group confirmed that blood cell lysis occurred in the PTX–Albumin group with 20 μg/mL of PTX. Meanwhile, PTX-GSSG-PEG with 20 μg/mL of PTX significantly decreased the hemolysis phenomenon compared with PTX–Albumin. These results demonstrate that PTX-GSSG-PEG could increase the biocompatibility of free PTX compared with the PTX–Albumin pharmaceutical.

## 4. Conclusions

In this study, a glutathione-responsive paclitaxel prodrug micelle was prepared based on a linker of an endogenous molecule of L-glutathione oxidized for cancer therapy using one-pot synthesis. The structure of PTX-GSSG-PEG was successfully characterized using NMR, UV-Vis, FT-IR, and MS. The particle size remained stable in PBS for 7 days, as measured using DLS. The accumulative drug release curve confirmed that the PTX-GSSG-PEG micelles showed a GSH-responsive drug release property. Cellular uptake experiments demonstrated that the micelles could be swallowed by MCF-7 cells and distributed into the cell nuclei. A CCK-8 assay and flow cytometry confirmed that the PTX-GSSG-PEG micelles showed similar drug efficacy to PTX–Albumin. Additionally, a hemolysis assay confirmed that PTX-GSSG-PEG could increase the biocompatibility of free PTX compared with PTX–Albumin. Together, the results demonstrate that this PTX-GSSG-PEG micelle can effectively inhibit the growth of tumor cells and have increased biocompatibility compared with PTX–Albumin. In conclusion, this micelle prepared based on a linker of endogenous molecules and an FDA-approved excipient of PEG has the potential for clinical application in the future.

## Data Availability

The original contributions presented in this study are included in the article/supplementary material, and further inquiries can be directed to the corresponding authors.

## Supplementary Materials

The following supporting information can be downloaded at: https://www.mdpi.com/article/10.3390/pharmaceutics16091178/s1, Figure S1: The mass spectrum of PTX-GSSG-PEG measured using MALDI-TOF; Figure S2: The elute time of (A) PTX-GSSG-PEG (16.305 min) and (B) PEG_5000_ (16.330 min) measured using gel permeation chromatography in THF.
