# Supplementary material for: Preparation of Glutathione-Responsive Paclitaxel Prodrug Based on Endogenous Molecule of L-Glutathione Oxidized for Cancer Therapy
Source: Pharmaceutics. 2024 Sep 6;16(9):1178. doi: 10.3390/pharmaceutics16091178 (PMC11435141; doi:10.3390/pharmaceutics16091178)
Supplement: Supplementary file 1 [file pharmaceutics-16-01178-s001.zip › pharmaceutics-3148356-supplementary.pdf]

Preparation of glutathione-responsive paclitaxel prodrug based on endogenous molecule of L-glutathione oxidized for cancer therapy

Xiao Duan [1,2,*,†], Qiang Wang [1,†], Yue Wang [3], Xinping Liu [1], Manman Lu [3], Zhifang Li [1], Xuelian Jiang [1] and Jingquan Ji [3,*]

[1] Changzhi Key Laboratory of Drug Molecular Design and Innovative Pharmaceutics, Shanxi Provincial Department-Municipal Key Laboratory Cultivation Base for Quality Enhancement and Utilization of Shangdang Chinese Medicinal Materials, School of Pharmacy, Changzhi Medical College, Changzhi 046000, China; wangqiang990326@163.com (Q.W.); xp99714@163.com (X.L.); 19834819324@163.com (Z.L.); 17860396873@163.com (X.J.)

[2] The Stem Cell and Tissue Engineering Research Center, Changzhi Medical College, Changzhi 046000, China

[3] Central Lab Changzhi Medical College, Changzhi Medical College, Changzhi 046000, China; 17696157396@163.com (Y.W.); 13383453636@163.com (M.L.)

* Correspondence: duanxiao0211@czmc.edu.cn (X.D.); jijingquan@czmc.edu.cn (J.J.)

† These authors contributed equally to this work.

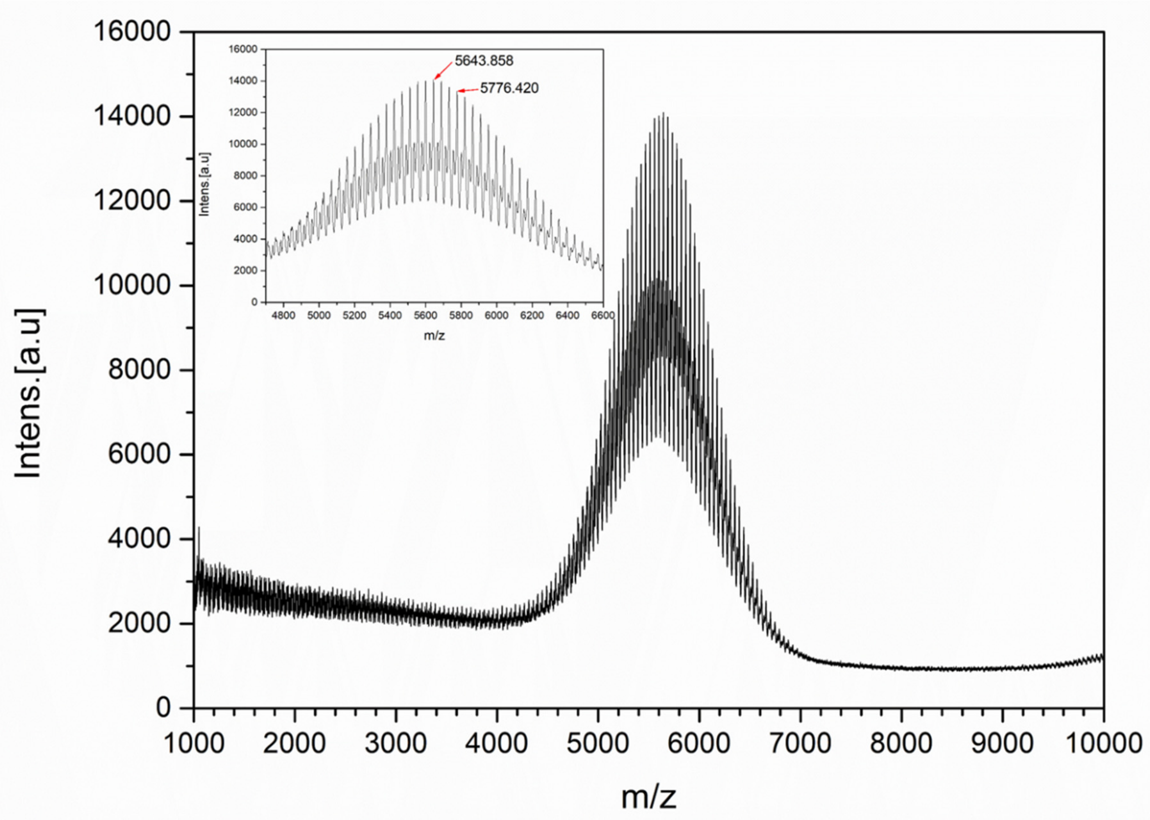

Figure S1 The mass spectrum of PTX-GSSG-PEG measured using MALDI-TOF

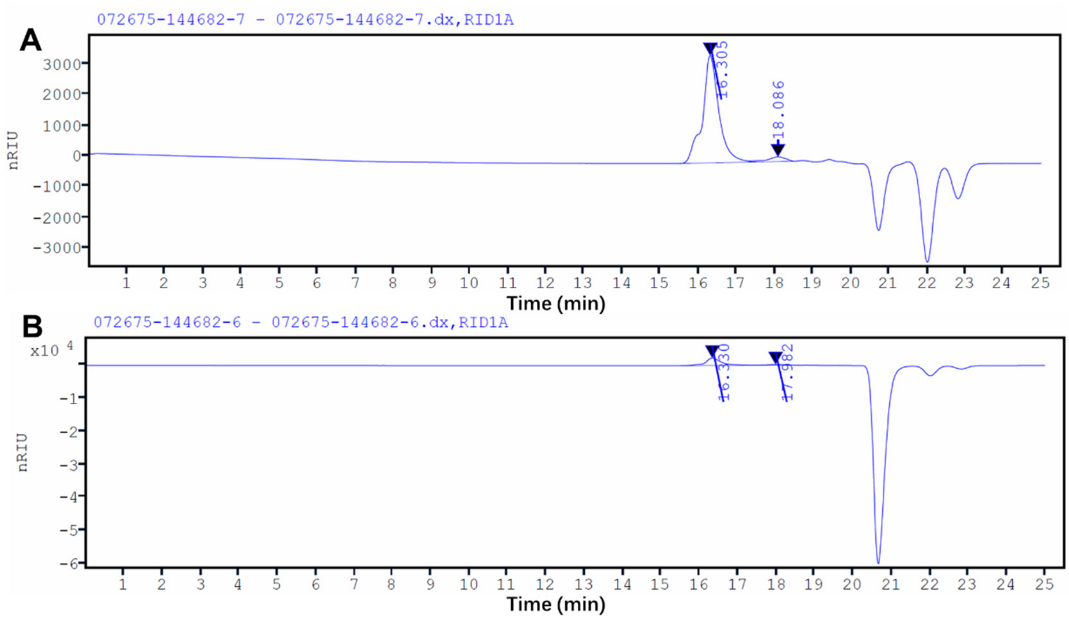

Figure S2 The elute time of (A) PTX-GSSG-PEG (16.305 min) and (B) PEG_{5000} (16.330 min) measured using gel permeation chromatography in THF
